# Correlation analysis of the side-chains conformational distribution in bound and unbound proteins

**DOI:** 10.1186/1471-2105-13-236

**Published:** 2012-09-17

**Authors:** Tatsiana Kirys, Anatoly M Ruvinsky, Alexander V Tuzikov, Ilya A Vakser

**Affiliations:** 1 Center for Bioinformatics, The University of Kansas, 66047, Lawrence, KS, USA; 2 United Institute of Informatics Problems, National Academy of Sciences, 220012, Minsk, Belarus; 3 Department of Molecular Biosciences, The University of Kansas, Lawrence, 66045, KS, USA

**Keywords:** Protein interactions, Protein docking, Molecular recognition, Conformational analysis

## Abstract

**Background:**

Protein interactions play a key role in life processes. Characterization of conformational properties of protein-protein interactions is important for understanding the mechanisms of protein association. The rapidly increasing amount of experimentally determined structures of proteins and protein-protein complexes provides foundation for research on protein interactions and complex formation. The knowledge of the conformations of the surface side chains is essential for modeling of protein complexes. The purpose of this study was to analyze and compare dihedral angle distribution functions of the side chains at the interface and non-interface areas in bound and unbound proteins.

**Results:**

To calculate the dihedral angle distribution functions, the configuration space was divided into grid cells. Statistical analysis showed that the similarity between bound and unbound interface and non-interface surface depends on the amino acid type and the grid resolution. The correlation coefficients between the distribution functions increased with the grid spacing increase for all amino acid types. The Manhattan distance showing the degree of dissimilarity between the distribution functions decreased accordingly. Short residues with one or two dihedral angles had higher correlations and smaller Manhattan distances than the longer residues. Met and Arg had the slowest growth of the correlation coefficient with the grid spacing increase. The correlations between the interface and non-interface distribution functions had a similar dependence on the grid resolution in both bound and unbound states. The interface and non-interface differences between bound and unbound distribution functions, caused by biological protein-protein interactions or crystal contacts, disappeared at the 70° grid spacing for interfaces and 30° for non-interface surface, which agrees with an average span of the side-chain rotamers.

**Conclusions:**

The two-fold difference in the critical grid spacing indicates larger conformational changes upon binding at the interface than at the rest of the surface. At the same time, transitions between rotamers induced by interactions across the interface or the crystal packing are rare, with most side chains having local readjustments that do not change the rotameric state. The analysis is important for better understanding of protein interactions and development of flexible docking approaches.

## Background

Protein-protein interactions play a key role in life processes. Characterization of conformational changes in proteins upon binding is important for understanding the mechanisms of protein association and for our ability to model it. Dependence of side-chain dihedral angle distribution on the conformation of the backbone has been investigated in earlier studies [1-5]. The side-chain dihedral angles are not evenly distributed, but for the most part are tightly clustered. A number of unbound rotamer libraries have been described previously [1-14] (see [15] for a review). Dunbrack and Cohen [1] used Bayesian statistics to estimate populations and dihedral angles for all amino acids rotamers at all φ and ψ values. A backbone-dependent rotamer library [15] was obtained by dividing φ and ψ dihedral space into 10°× 10° bins, χ angles into 120° bins, and calculating frequencies and average values of rotamers for each amino acid. A backbone-independent rotamer library was generated in a similar way. In a recent study [16], a new version of the backbone-dependent rotamer library was developed. It consists of rotamer frequencies, mean dihedral angles, and variances as a function of the backbone dihedral angles. In one of the latest backbone-independent rotamer libraries, the “Penultimate rotamer library” [5] by Lovell, Richardson and colleagues, the dihedral angle space was clustered and rotamer positions were defined as the distribution mode.

Comparison of the side-chain distribution in the core and on the surface [3], conducted on 19 protein structures available in 1978, revealed a small variation of the χ_1_ rotamers distribution. A later study [17] on a set of 50 non-homologous proteins showed that for all side chains, except Asp, Asn and Glu, the distributions of χ_1_ rotamers on the surface and in the core are not significantly different.

Comparison of the χ_1_ and χ_2_ distributions at the interface and non-interface surface was performed by Guharoy et al. [18]. Distributions were divided into bins as in the Dunbrack’s backbone-independent rotamer library [1]. Empirical free energies of inter-rotamer transitions were calculated and compared for the interface and non-interface areas. The rotamers free energies were different at the interface and non-interface, whereas bound and unbound free energies were essentially the same.

Conformations of surface residues in protein structures determined by crystallography are affected by the crystal packing. The area of the protein surface involved in the crystal contacts is generally smaller than in biological interfaces [19], and the interface packing is looser [20]. Studies of the crystal packing effect on the surface side chains [21-23] showed that ~ 20% of the exposed side chains change conformation, and the change increases with the increase of the side-chain solvent accessibility. Large polar or charged residues Arg, Lys, Glu, Gln, as well as Ser were found to be most flexible [21].

The purpose of this study was to analyze and compare dihedral angle distribution functions of the side chains at the interface and non-interface areas in bound and unbound proteins. Such analysis is important for better understanding of protein interactions and development of flexible docking approaches. The dihedral-angle distribution functions (DADF) were calculated on a cubic grid dividing the dihedral space into cells for each residue type, at interface and non-interface surface, in bound and unbound structures. The correlation coefficients between bound and unbound, interface and non-interface DADFs were calculated, along with the Manhattan distance, as a measure of dissimilarity between the DADFs. All the correlation coefficients depended on the amino acid type and the grid resolution. The correlation coefficients always increased with the increase of the grid spacing, whereas the Manhattan distances decreased accordingly. Short residues with one or two dihedral angles had higher correlations and smaller Manhattan distances at small grid spacing than the longer residues. The correlation between the interface and non-interface DADFs showed a similar dependence on the grid resolution in both bound and unbound states. The differences between bound and unbound DADFs induced by biological protein-protein interactions or crystal contacts disappeared at the 70° grid spacing for interfaces and 30° for non-interface surface. The two-fold difference in the critical grid spacing indicates larger changes at the interface than on the rest of the surface. While the earlier studies [18,24,25] observed this trend for the side-chain rotamers, this study validates it by a more general approach based on the DADFs.

## Methods

The analysis was performed on the non-redundant Dockground Benchmark 3 set of bound and corresponding unbound protein structures [26]. The set consists of 233 complexes, with the unbound structures of both interacting proteins for 99 complexes, and the unbound structure of one interacting protein for 134 complexes. The following criteria were used for generating the set: sequence identity between bound and unbound structures > 97%; sequence identity between complexes < 30%; and homomultimers, crystal packing, and obligate complexes excluded.

The core residues change conformation upon binding less than the surface ones [24]. Thus, our study focused on the surface residues only. Surface residues were defined as those with the relative solvent-accessible surface area ≥ 25% in bound and unbound state. The change of the residue solvent-accessible surface area (SASA) upon binding was used to differentiate the interface residues from the non-interface ones. SASA was calculated using Naccess [27]. The interface residues were defined as those losing > 1 Å^2^ SASA upon binding. The statistics of the interface and non-interface residues in the bound and unbound structures are summarized in Table 1. The difference between the numbers of bound and unbound interface/non-interface residues reflects the difference between the number of bound and unbound protein structures in the Dockground set.

**Table 1:** Number of surface residues in bound and unbound proteins

**Amino acid**	**InterfaceU**^**a**^**/B**^**b**^	**Non-interface U/B**
Ser	333/429	2409/2934
Val	175/259	1003/1116
Thr	321/406	2021/2375
Cys	40/58	183/190
Pro	250/295	1727/2029
Ile	136/202	560/638
Leu	245/348	1095/1258
Asn	291/350	1902/2074
Asp	329/433	2448/2856
His	126/174	682/741
Phe	108/176	457/487
Tyr	202/296	636/718
Trp	63/100	187/220
Gln	268/357	1687/1888
Glu	416/545	2933/3303
Met	73/119	311/329
Lys	427/555	2965/3387
Arg	325/474	1791/2014

^a^Unbound.

^b^Bound.

Side chain conformations were represented by dihedral angles, calculated by Dangle [28]. All dihedral angles varied from −180° to 180°, with exception of the last dihedral angle in Phe, Tyr, Asp and Glu [2], which varied from 0° to 180° due to the symmetry of the terminal aromatic and charged groups. To calculate the distribution functions, the configuration space was divided into cells by a cubic grid.

**Figure 1:**
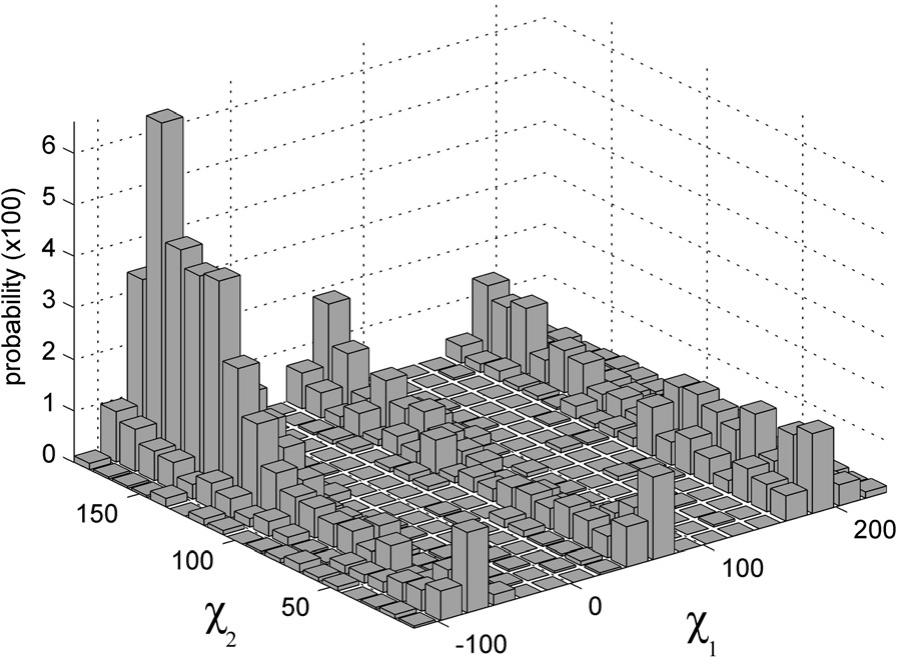
Dihedral angle distribution of non-interface Asp in unbound structures.

DADFs were calculated as the occupancy of the grid cells separately for each residue type for interface and non-interface, bound and unbound residues. Thus, there were four DADFs for each residue type: interface bound, interface unbound, non-interface bound, and non-interface unbound. Figure 1 shows a two-dimensional distribution function of Asp dihedral angles for the non-interface unbound residues.

To compare distributions X and Y, the corresponding n-dimensional space (n is the number of the dihedral angles in the side chain) was split into m cubes with a fixed side length. The occupancy in each cell was calculated (Figure 1). The correlation coefficient r [29] between unbound (X) and bound (Y) DADFs was calculated as:

(1) $$r=\frac{\sum_{i=1}^{m}\left(X_{i}-\bar{X}\right)\left(Y_{i}-\bar{Y}\right)}{\sqrt{\sum_{i=1}^{m}{\left(X_{i}-\bar{X}\right)}^{2}}\sqrt{\sum_{i=1}^{m}{\left(Y_{i}-\bar{Y}\right)}^{2}}}\text{,}$$

where X_i_ and Y_i_ are the probabilities of bound and unbound side-chain conformations in a grid cell i, $\bar{X}=\frac{1}{m}\sum_{i=1}^{m}X_{i}$ and $\bar{Y}=\frac{1}{m}\sum_{i=1}^{m}Y_{i}$ are the average probabilities of bound and unbound side-chain conformations. To determine the degree of similarity between two probability distributions the Manhattan distance [30] was calculated as:

(2) $$d\left(X,Y\right)=\frac{1}{2}\sum_{i=1}^{m}\left|X_{i}-Y_{i}\right|$$

The Manhattan distance equals 0 for two identical DADFs, and increases up to 1 with the decrease of the DADFs similarity (higher similarity between the DADFs corresponds to lower values of the Manhattan distance).

## Results and discussion

**Figure 2:**
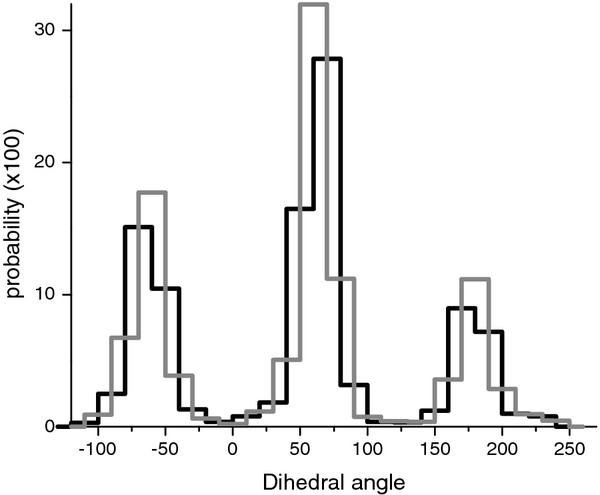
Dihedral angle distribution of non-interface unbound Ser with 20° grid spacing and different splitting points.

The discrete probability distribution of the amino acid side-chain χ angles depended on the starting point of splitting and the size of the grid spacing. An example of a probability function with 20° grid spacing and different starting points of splitting for non-interface unbound Ser is shown in Figure 2. The distribution was divided into cells with a predefined step size, starting with a randomly chosen point, and the probability in each cell was calculated. To remove the effect of splitting, correlation coefficients were calculated 100 and 1000 times with the same splitting step but random starting point of splitting. Then, the average correlation coefficients were calculated. We found no significant difference between the correlation coefficients averaged 100 or 1000 times. Tests of statistical significance of the correlation [31] between bound and unbound distributions, and non-interface and interface distributions showed that all correlation values were significant, with p-values far below 0.001.

**Figure 3:**
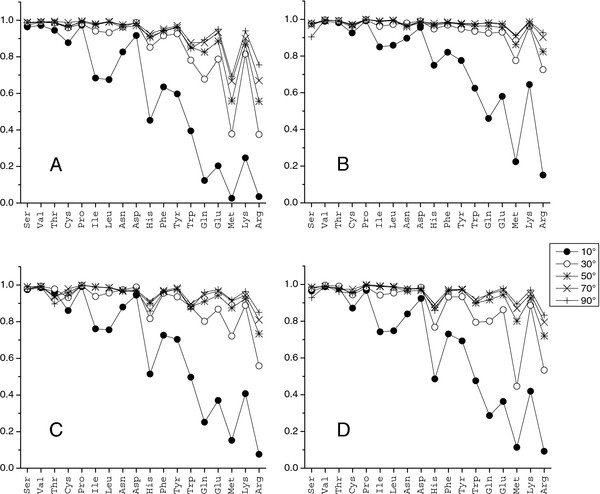
**Correlation between dihedral angle distributions.** (**A**) Interface bound vs. unbound, (**B**) non-interface bound vs. unbound, (**C**) non-interface vs. interface unbound, and (**D**) non-interface vs. interface bound. For each grid spacing, 100 tests were performed with random splitting point. The plot shows the average correlation value.

Analysis showed that the correlation coefficients depend on the grid spacing (Figure 3). Generally, larger steps corresponded to higher correlation values (larger cells yielded more smooth/similar distributions). Table 2 shows the grid spacing at which the correlation reaches a high level of 0.7. Most amino acids had high correlation between bound and unbound interface/non-interface distributions for grid spacing ≤ 20°, except Met and Arg at the interface and non-interface, and Glu and Gln at the interface. The correlation coefficient for Met and Arg increased with the grid spacing increase and reached the high level of 0.7 at the 70° grid spacing for interface, and 30° for non-interface. The two-fold difference in the critical grid spacing indicates higher flexibility of these amino acids at the interface [24]. Since the 120° distance between two adjacent side-chain rotamers is significantly larger than the critical grid spacing, the use of large clustering radii for bound and unbound rotamer libraries [24] would produce similar results.

**Table 2:** The minimal grid spacing corresponding to correlation coefficient 0.7 between bound and unbound interface/non-interface dihedral angle distribution

**Amino acidNI**^**a**^	**stepNI**^**a**^	**I**^**b**^
Ser	10	10
Val		
Thr		
Cys		
Pro		
Ile		
Leu		
Asn		
Asp		
His		20
Phe		
Tyr		
Trp	20	
Gln		30
Glu		
Met	30	70
Lys	20	20
Arg	30	70

^a^Non-interface.

^b^Interface.

Although the results showed high degree of similarity between the distributions, correlation values for Met and Arg were noticeably lower than for other amino acids. Analysis of the results for Met revealed that although the covariance of distributions for all amino acids with three dihedral angles were the same, the standard deviation for Met was higher (Table 3), leading to the lower correlation value for Met. In the case of Arg, although the standard deviations of Lys were twice larger than that of Arg, the covariance of Arg was ten times smaller than that of Lys, yielding the overall lower correlation for Arg.

**Table 3:** Correlation between interface bound and unbound distributions for 30° grid spacing

**Amino acid**	**Covariance (numerator in Equation1)**	**Product of Standard deviations (denominator in Equation 1)**	**Standard deviations of the unbound DADF**	**Standard deviation of the bound DADF**	**Correlation**
Ser	0.0830	0.0839	0.2884	0.2910	0.9892
Val	0.1414	0.1456	0.3862	0.3769	0.9714
Thr	0.1217	0.1229	0.3475	0.3536	0.9906
Cys	0.1162	0.1259	0.3315	0.3797	0.9235
Pro	0.1681	0.1725	0.4148	0.4158	0.9744
Ile	0.1002	0.1048	0.3446	0.3042	0.9561
Leu	0.1343	0.1358	0.3486	0.3896	0.9891
Asn	0.0302	0.0329	0.1815	0.1815	0.9174
Asp	0.0313	0.0338	0.1727	0.1959	0.9242
His	0.0358	0.0394	0.1944	0.2026	0.9101
Phe	0.0520	0.0577	0.2201	0.2621	0.9020
Tyr	0.0449	0.0477	0.2058	0.2317	0.9415
Trp	0.0335	0.0419	0.2174	0.1927	0.8003
Gln	0.0056	0.0093	0.0901	0.1030	0.5984
Glu	0.0069	0.0089	0.0900	0.0986	0.7770
Met	0.0071	0.0194	0.1403	0.1383	0.3676
Lys	0.0096	0.0116	0.1067	0.1092	0.8286
Arg	0.0012	0.0033	0.0535	0.0620	0.3515

**Figure 4:**
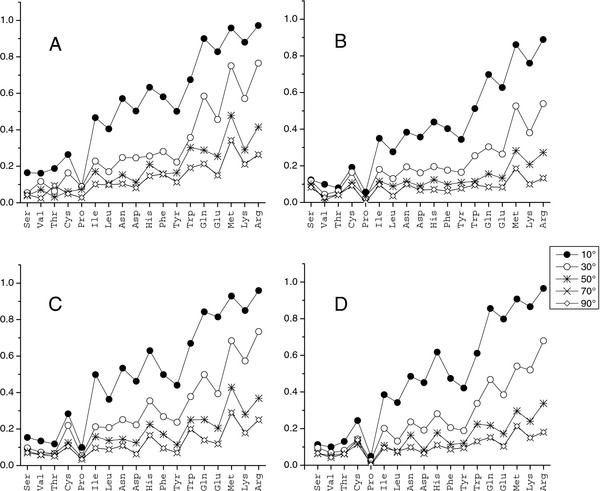
**Manhattan distance between dihedral angle distributions.** (**A**) Interface bound vs. unbound, (**B**) non-interface bound vs. unbound, (**C**) non-interface vs. interface unbound, (**D**) non-interface vs. interface bound.

**Figure 5:**
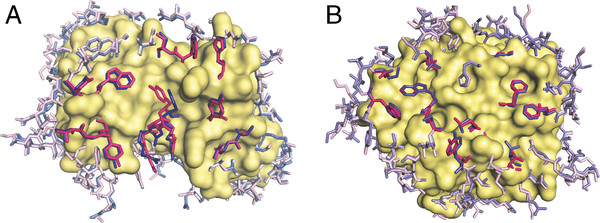
**Examples of side-chain conformational changes upon binding.** (**A**) Immunoglobulin and (**B**) alpha-chymotrypsin in the unbound (blue) and bound (magenta) states. The core residues are shown as surface. The interface residues are shown in bold colors. The bound structure of the immunoglobulin is 1a2y [32], the unbound structure is 1vfa [33]. The bound structure of the alpha-chymotrypsin is 1acb [34], and the unbound structure is 1gct [35].

Equation 2 was used to calculate the Manhattan distance between bound and unbound interface/non-interface distributions. As in the case of correlation, the metric value depended on the grid spacing, with larger steps corresponding to more coarse-grained distributions. Thus, tests were conducted with different steps: 10°, 30°, 50°, 70°, and 90°. The distance between the distributions decreased with the step increase (Figure 4). In most cases, the Manhattan distances for the interface were greater than for the non-interface. The distances between interface unbound and bound distributions for all long amino acids with three and four dihedral angles were the largest (Figure 4A). It agrees with our previous findings that long amino acids have higher flexibility in binding [24]. The Manhattan distance between the probability functions was < 30% for most amino acids, starting with 50° grid spacing, except for Met and Arg interface bound vs. unbound and non-interface vs. interface distributions. For these distributions, the distance was < 30% at grid spacing 70°, and < 35% for Met interface bound vs. unbound and Arg bound non-interface vs. interface. The high similarity between the DADFs at the 50° grid spacing is a result of the small number of rotamer-to-rotamer transitions induced by interactions across the interface or the crystal packing. Most side chains have local readjustments (Figure 5) that do not change the rotameric state.

## Conclusions

The dihedral-angle distribution functions were calculated for each amino acid type for interface and non-interface surface residues, in bound and unbound protein structures. To generate the distribution functions, the configuration space was divided into cells by a cubic grid. Correlation coefficients between bound and unbound interface and non-interface distribution functions were calculated. The similarity between the distributions was also quantified by the Manhattan distance. The results showed that all the correlation coefficients depend on amino acid type and the grid resolution. For all amino acid types, the correlation coefficients increased with the increase of the grid spacing. The Manhattan distances between the distribution functions decreased accordingly. Short residues with one or two dihedral angles had higher correlations and smaller Manhattan distances than the longer residues. Met and Arg had the lowest correlation coefficients at any grid spacing. The correlations between the interface and non-interface distribution functions had a similar dependence on the grid resolution in both bound and unbound states. The interface and non-interface difference between bound and unbound distribution functions, induced by biological protein-protein interactions or crystal contacts, disappeared at the 70° grid spacing for interfaces and 30° for non-interface surface, in agreement with an average span of a side-chain rotamer. The two-fold difference in the critical grid spacing indicates larger conformational changes upon binding at the interface than at the rest of the surface. At the same time, transitions between rotamers induced by interactions across the interface or the crystal packing are rare, with most side chains having local readjustments that do not change the rotameric state.

Conformational sampling based on the side chain dihedral angle distributions may optimize flexible docking protocols by reflecting conformational preferences of the bound proteins. The results suggest that the site- (interface vs. non-interface) and residue-specific grid spacing smaller than the critical values should be used in the sampling. The minimal grid spacing (Table 2) reflects intra-rotamer amino acid local readjustments upon binding. Thus, using such steps in conformational sampling may accelerate the flexible docking search by reflecting the size of these readjustments.

## Competing interests

The authors declare that they have no competing interests.

## Authors' contributions

All authors conceived and designed the research. TK and AMR carried out the calculations, and all authors analyzed the results. The manuscript was drafted by TK and written/revised by all authors, who read and approved the final manuscript.

## Authors' information

TK is a PhD student at the United Institute of Informatics Problems, National Academy of Sciences of Belarus and a Research Assistant at the Center for Bioinformatics, The University of Kansas; AMR is an Assistant Research Professor at the Center for Bioinformatics, The University of Kansas; AVT is the General Director of the United Institute of Informatics Problems, National Academy of Sciences of Belarus; and IAV is the Director of the Center for Bioinformatics and Professor of Bioinformatics and Molecular Biosciences at The University of Kansas.
